# The “heterogeneous” effect of government grants on bank lending

**DOI:** 10.1371/journal.pone.0289375

**Published:** 2023-12-11

**Authors:** Shuibin Gu, Qinyue Zhang

**Affiliations:** 1 School of Finance and Economics, Jiangsu University, Zhenjiang, Jiangsu, China; 2 WHU-DUFE Management Control Research Center, Dongbei University of Finance and Economics, Dalian, Liaoning, China; Shanghai Business School, CHINA

## Abstract

This study aims to test whether banks can recognize different signals from different types of government grants received by their clients and respond differently. Using a novel panel data set from China, we construct a three-way fixed-effect regression model and empirically explore the effect of government grants received by banks’ clients on banks’ lending decisions. We find that banks have heterogeneous attitudes towards their clients when the clients receive different types of government grants. In particular, we discover that banks behave positively toward clients who receive "development supportive" grants but negatively toward clients who receive "helping hand" grants. The main results hold after a series of robustness tests. Our study offers fresh perceptions on how banks see government grants of their clients while making lending choices. Our finds will offer certain inspirations for government grant policies and bank lending decisions.

## 1 Introduction

Government grants are frequently employed as a tool for policy to intervene in the market and counteract market failures [1, 2]. Government grants have been shown in prior research to have both "subsidy effects," such as providing funding to correct the positive externalities of businesses’ research and development [3], and "signal effects," such as reducing information asymmetry and enhancing financing capabilities [4, 5].

Most earlier research views receiving government grants as a positive indicator for external funding. For instance, one study [6] discovers that obtaining bank loans for subsidized businesses is simpler. Another study [5] argues that government grants can make it easier for subsidized firms to acquire lower debt financing. We contend, however, that government grants may also contain a negative signal and constrain external financing.

We consider that government grants, in our opinion, should be divided into two categories: "development supportive" and "helping hands" [7, 8]. Different types of government grants take place for different purposes and will send different signals to the market. "Development supportive" grant is inherently intended for the government to assist companies in specific new industries to grow or develop. The “Helping hands” grant is often used by the government to help financially distressed companies to survive. In contrast to the latter, which is contingent, sudden, and unsustainable, the former has some continuity, is sustainable, and is typically taken as operating profit. Failing to recognize the difference between these two kinds of government grants may lead to wrong decisions. Government grants have been extensively studied, but few notice the difference in different government grants. Little is known about whether such grants may have heterogenous effects [9–11]. Our research aims to fill this gap in the literature.

This study aims to discover whether banks can recognize different types of government grants and respond differently. We concentrate on banks because they are dominant external funding providers in most of the world’s nations [12]. Banks may make capital misallocation decisions and thus cause systematic financial risks if they cannot accurately discern the signals of the government grants received by their clients. Therefore, our research aims to determine if banks can react appropriately to various government grants.

We anticipate that two types of government grants will have differing effects on banks’ lending decisions. First, when their clients receive "development supportive" grants, we anticipate banks to act positively by offering affordable financing. Second, we anticipate that banks will adopt a pessimistic attitude when their clients receive "helping hands" grants by offering less and more expensive financing. Those subsidies can increase the suspicion of clients’ poor performance.

**Fig 1:**
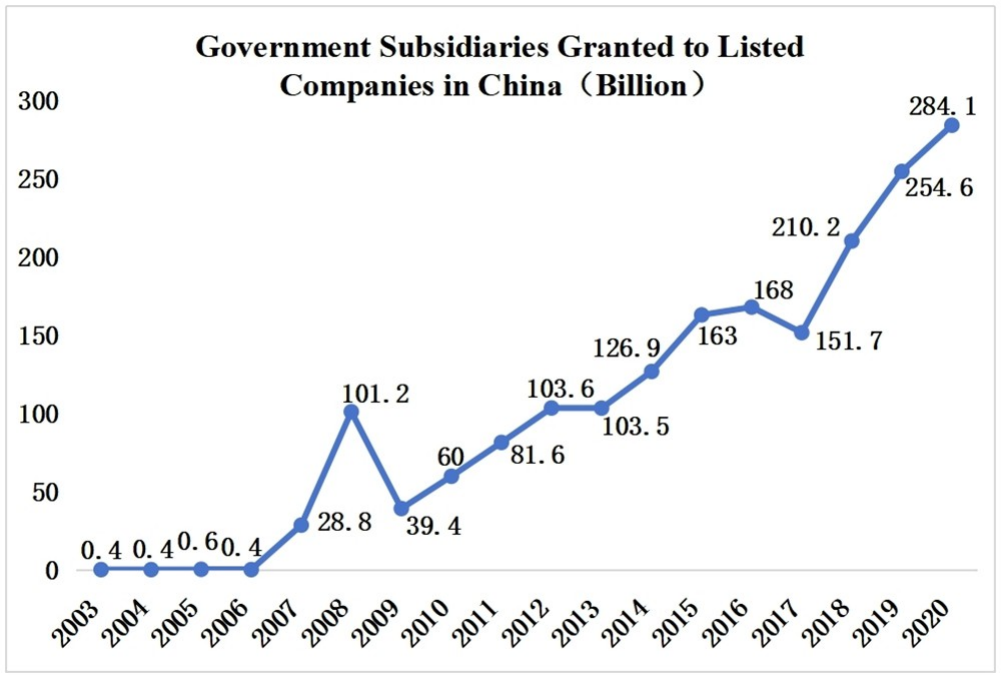
Government grants granted to Chinese listed companies from 2003 to 2020.

To test hypotheses, we employ a unique setting from China. The firm-level government grant data is not publicly available in most countries, where disclosure requirements on grants are absent [13]. However, government grant recipients in China must disclose grant details in financial statements on time. These disclosure requirements offer us an opportunity to investigate our question closely. Besides, an exciting feature of China that distinguishes it from other peers is the pervasiveness of government grants, which makes another good reason for us to use a Chinese sample [5, 7] (Fig 1). The government grant in China represented up to 20% of the average operational profits in 2019 and 2020, covering 98.58% and 99.25% of all Chinese listed companies, respectively [14]. We will benefit from China’s extensive range and scope of government grants as we observe banks’ responses from multi-perspectives.

Based on 15408 firm-year observations from 2009 to 2019, we document evidence that banks have asymmetries in their sentiments toward government grants received by their clients. Banks only have a positive attitude toward "development supportive" grants and a negative attitude toward "helping hands" grants. Banks lend more money to clients who receive "development supportive" grants and charge higher interest to clients who receive "helping hand" grants. The cross-sectional results increase our confidence that the documented heterogeneous attitudes of banks are due to the various types of government grants received by their clients.

To further test the mechanisms, we take full use of the government grants presentation. We employ two different financial presentations to measure two types of government grants respectively. According to our findings, banks provide more financing and charge lower interest rates for "development supportive" grants but provide less financing and charge higher interest rates for "helping hands" grants.

Our study makes significant literary contributions. First, our study will guide future research to pay attention to the difference in government grants and their various effects. To the best of our knowledge, this is the first study to examine the heterogeneity in effects toward various types of government grants. The majority of the prior research ignores differences in various government grants and fails to model the underlying dynamics. Our study varies from earlier studies in that we propose a new framework and offer some evidence that banks’ attitudes change in response to different types of government grants received by their clients. One study highlights the mechanism underlying the heterogeneous effects of government grants and their various effects have yet to receive enough attention [15].

Second, this paper suggests that the size and cost views should simultaneously be used to evaluate lending attitudes. Cost and size are two distinct factors. To accurately reflect banks’ lending attitudes, these two factors must complement each other. Lending cost captures how much to charge after the lending is granted while lending size captures if and how much the lending should be approved. Prior research has mostly examined banks’ attitudes from a single perspective of size or cost, which does not provide the full picture and may lead to wrong conclusions [4, 13].

Third, to our best knowledge, we are the first to use financial presentations to categorize and measure government grants. Prior literature, due to the lack of uniform disclosure format and classification standards for government grants, mainly uses total government grants or textual analysis to measure [5, 16]. The inaccurate measurements of government grants may lead to biased conclusions.

The remainder of the paper is structured as follows. Section 2 presents a theoretical framework and hypotheses. Section 3 describes the variables, models, and samples. Section 4 reports the main findings. Sections 5 and 6 provide a mechanism analysis. The paper ends with a discussion of the findings, policy implications, and potential avenues for future research.

## 2 Theoretical analysis and hypotheses development

### 2.1 Theoretical analysis

Under classic economics, a lending contract is assumed to be written under the condition that banks know their clients well, although this is not the case. As there is always information asymmetry between them, bank lending is like an investment in an uncertain commodity. Banks, in this sense, are risk averters, unlike investors. Self-interested credit managers are typically cautious and diligent agents who risk losing their jobs if they make mistakes. They must, however, lend enough money to help banks make profits. Thus, they cannot be overly cautious or conservative. Therefore, they have a strong motivation to scrutinize, monitor and pay close attention to any material information pertaining to their clients to back up their lending decisions.

Bank lending decision is a complex psychological process. Banks usually provide different contracts to their clients concerning the type. The cognitive process of forming a judgment or making a decision on loans with financial data typically serves us very well since it is effortful, explicit, and logical. Even so, there are times when sole financial data may lead us to the wrong conclusion or outcome. One study argues that traditional lending practices that rely primarily on financial data may cause lending decisions to be biased because financial data only provides a partial image of a company [17]. Another study finds that non-financial information collected through observation and third parties contributes to framing the credit manager’s perception of a firm and plays an essential role in complementing financial data [18].

**Fig 2:**
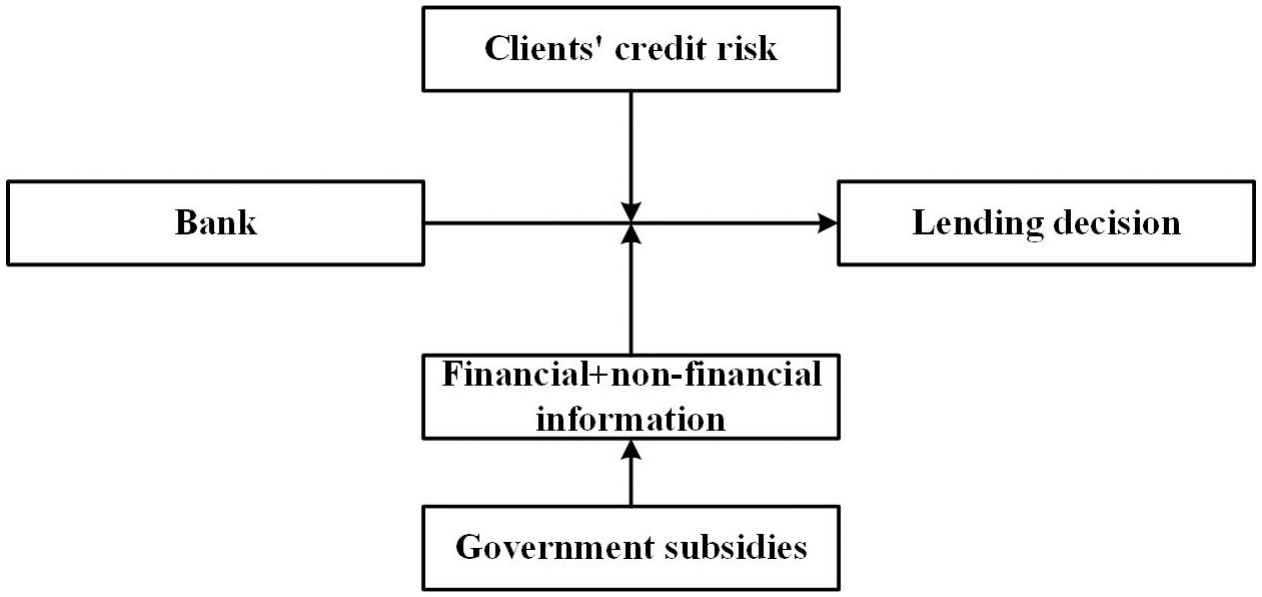
Framework of government grants on lending decision.

According to the signaling theory, receiving government grants can be translated into signals and cause effects either explicitly through mandated reporting or implicitly through the signal provided by the grants’ acceptance, the media, and some watchdog organizations [19, 20]. Receiving government grants can contain valuable information, as the government’s decisions are not entirely random. To receive government grants, a firm needs to file an application that will be reviewed at various levels of government. Governments play an essential role as gatekeepers and referees of firms’ applications for government grants. The approval of government grants can send specific signals to the market, including the firm’s type and other information, which could help complete credit managers’ perception of the things that they might know little about their clients. Besides financial information, receiving government grants could be another critical information source. The illustrated principle is in Fig 2.

### 2.2 Hypothesis development

As argued in the seminal paper [21], they find that a high failure rate of R&D makes it difficult for the external market to evaluate the quality of the firms, and thus exacerbates the financing constraints and scales down the investment in R&D. Government grants could play a screening signal for good investment projects that can reduce information asymmetry, help firms improve their image and increase their access to resources. Especially early-stage startups in emerging industries may even be challenging to signal themselves. However, the government could utilize government grants as “a visible hand” to help send signals to the market. Government grants could reduce the information asymmetry between businesses and the market and assist businesses in attracting venture capital [21] and securing bank loans [22].

In addition, government grants may also help firms to get more access to bank loans via credit endorsement. The receipt of government grants could carry a certification signal to investors that the receiving firm has good prospects and less uncertainty since it has government backing [23]. Government grants may suggest to investors that subsidized companies have better prospects and less uncertainty because the government supports them [7]. One study [24] finds that the signal of government grants has a certain endorsement effect, facilitating firms’ access to external financing. The other study [25] finds a positive certification effect on acquiring bank loans for all those sample firms. Another study [13] finds that government grant is an implicit guarantee, and firms with more government grants usually have lower debt costs. In a different study [26], authors find that government grants primarily serve as a certificate for political capital rather than the quality of the firms in China.

In essence, receiving government grants could be interpreted into visible, measurable, and less manipulable signals sent to the market. These signals could boost companies’ credit endorsement to the market and decrease information asymmetry between firms and the market [27]. Thus, we propose our first set of hypotheses:

H1a: Ceteris paribus, banks would like to lend more money to high-subsidized firms.
H1b: Ceteris paribus, banks would like to charge cheaper interest to high-subsidized firms.

The government’s role in economies is still a topic of discussion [7]. Government and the market both follow separate rules of operation. While government favours intervention and planning, the market promotes competition. The invisible hand (pricing and competition) may not function well when there is government interference. Government grants are crucial "helping hands" in the transition economy by lowering the threshold to entry for industry and investment, which may result in an imbalance between price and cost. One study [28] finds that government grants stimulated enterprises to make "imitative innovation" through patent purchase rather than independent R&D. Other studies [29–31] find that government grants can result in zombie companies and are most frequently seen in overcapacity industries including steel, cement, and coal.

Furthermore, receiving government grants in China must meet two criteria: poor performance and potential delisting. The government typically offers underperforming businesses "helping hands" to keep them from being delisted to maintain local GDP and a consistent employment rate. According to one study [32], authors find that governments may use subsidies to intervene in the market and achieve a specific employment rate. Numerous studies show that government favours subsiding underprivileged businesses [13]. Government grants are increased for businesses about to lose [16]. Government grants are given to businesses to help them overcome capital constraints and to support them when they are having financial difficulties [33]. One study [34] even discovers that subsidized companies aggressively smooth their earnings compared to unsubsidized companies. Another study [35] finds that government grants negatively affect the total factor productivity of businesses and induce unproductive rent-seeking activities rather than productive rent-seeking activities.

In conclusion, government grants may send the market unfavourable signals like "bad performance" and "possible delisting." Government grants provided to struggling businesses may artificially boost reported earnings and mask true losses, which could skew reported earnings and cause wrong conclusions. Additionally, political ties may weaken with time and government budgets may prevent government grants from being sustained [9]. As a result, banks should be cautious when making loan choices and may ask for a higher risk premium when a company receives excessive government funding. According to one study [36], government grants considerably raise the cost of debt.

H2a: Ceteris paribus, banks would like to lend less money to high-subsidized firms.
H2b: Ceteris paribus, banks would like to charge higher interest to high-subsidized firms.

**Fig 3:**
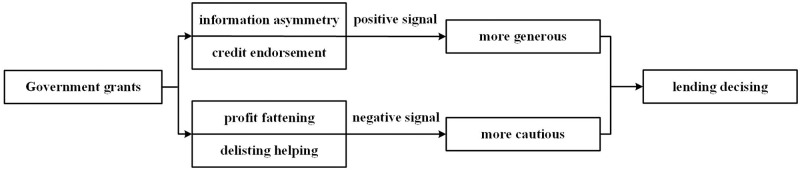
Signal effect of government grants on bank lending.

The above hypothesis can be illustrated in Fig 3.

## 3 Sample selection and research design

### 3.1 Sample and data sources

As government grants were high during the global financial crisis in 2008 and the COVID-19 pandemic from 2020, we select our sample spanning from 2009 to 2019. With the following exceptions, all A-share listed companies on the main board of the Shanghai and Shenzhen Stock Exchange in China were involved. All Specially Treated or suspended listing firms are first removed. Second, we exclude companies from the finance and insurance industries. Third, we eliminate the businesses with major variable values missing. Fourth, we eliminate companies with negative cash flows from borrowing or government grants. Finally, we have 15,408 firm-year observations. The data was gathered in the China Stock Market & Accounting Research Database (CSMAR). Complete financial information about the companies, bank loans, information on the type of grants, the grant year, and other facts are all provided by CSMAR. Table 1 summarizes the sample selection procedure.

**Table 1:** Sample selection.

Sampling Procedure	Observations
All observations from 2009 to 2019	20, 710
Less: observations of ST companies	1, 897
Less: financial and insurance companies	1, 320
Less: observations with missing data	975
Less: observations with negative government grants	123
Less: observations with negative cash flow from borrowing	987
Final observations	15, 408

### 3.2 Model specification

In the primary research, we look at how banks react to government grants received by their clients. We use the following three-way fixed-effect regression model in conjunction with prior studies [5, 13] to test. The variables are demonstrated below.

$${{Loan}_{i,j,t}(Cost}_{i,j,t})=\alpha_{0}+\alpha_{1}{Sub}_{i,j,t}+\alpha_{2}{PPE}_{i,j,t}+\alpha_{3}{Grow}_{i,j,t}+\alpha_{4}{Lev}_{i,j,t}+\alpha_{5}{Size}_{i,j,t}+\alpha_{6}{Roa}_{i,j,t}+\alpha_{7}{Own}_{i,j,t}+\alpha_{8}Firm\;FE+Year\;FE+Industry\;FE\;+\varepsilon_{i,t}$$ (1)

Where i indicates the firm, j represents the industry, and t denotes the time.

The dependent variables are lending size (*Loan*) and cost (*Cost*). While the second evaluates the lending risk, the first highlights whether banks should lend money. Missing one of those measures could result in wrong conclusions because the two measurements come from different perspectives and are incomparable. Some scholars [5] use debt ratio as a measure of lending size, however, this indicator is static and cannot capture changes in bank lending. Additionally, as that indicator disregards the offset between loan repayments and new borrowings, it can result in measurement errors. Due to this, we use the statement of cash flows’ "cash received from borrowing" data to measure bank lending and adjust it for total ending assets.

Our second dependent variable is lending cost. In line with prior literature [13], we use the ratio of interest expense to total interest-bearing liabilities to measure it.

Government grant (*Sub*) is our independent variable. Government grants are provided in various forms. Some of them may not report in financial statements. Our research focuses on those reported in financial statements. Some scholars [4] use a log–log specification, such as the natural logarithm of total government grants plus 1. We argue that the natural logarithm could cause heteroskedasticity in government grants to some extent, but it cannot assess the intensity of subsidies. Therefore, we use government grants divided by total assets to measure *Sub*. This treatment can overcome heteroskedasticity issues and help maintain a consistent deflator with dependent variables.

We note that firm characteristics, specific industries, and year factors may influence government grants and bank lending in the same time, which may cause endogeneity issues and biased coefficients. As a result, we use a set of control variables based on prior research [5, 37]. We first adjust for the characteristics of the firm. State, 1 if the business is owned by the state, 0 otherwise. The main reason is that state-owned companies have closer ties to the government and banks. Size is the total assets’ natural logarithm. According to "scale discrimination," the government is more likely to subsidize big firms to further its political goals. Large businesses receive most subsidies [38]. Larger businesses find them easier to borrow from banks and have a lower default risk. Net profit scaled by total ending assets is known as ROA. Profitability is an essential metric for bank lending and government grant. Profitability can be a proxy for both the ability to make money and the incentives for managing earnings. Growth is the increase in sales revenue from year to year. Businesses at various stages have varying capital requirements and will receive varying levels of government grants. Leverage is total liabilities divided by total assets. Leverage is a crucial component of bank financing. PPE is the total of inventories and fixed assets scaled by assets. Businesses with more properties may offer more collateral, which lowers the risk for the lenders. As needed for standardization, our control variables are scaled by total ending assets.

All continuous variables are winsorized at the 1st and 99th percentiles to guarantee that outliers do not influence our conclusions. All variables are defined in S1 Appendix.

### 3.3 Method selection

As we employ a set of panel data, we have to select a method from pooled OLS (ordinary least square), fixed effect and random effect [39]. According to classical econometrics, Pooled OLS regression models may produce biased estimates because they cannot address omitted variable bias. This type of bias could become an endogenous problem when the omitted variable that influences independent variables are correlated with the dependent variables, exerting either upward or downward bias. To overcome the potential bias, scholars suggest the use of fixed-effect models. Fixed-effect models address issues of bias by while holding invariant unobserved characteristics constant or fixed. Specifically, a fixed-effect model has a different constant value for each firm which provides a control for those factors that are permanent features of firms but are not explicitly examined in the statistical model. The fixed-effect model is estimated by regressing the differences in dependent variables on the differences in independent variables and other variables. Therefore, the constant and permanent factors drop out of the equation [40].

In our sample, we have some tests and detected the firm-fixed effect. We, therefore, control for cross-sectional variation in firm factors that influence banks’ lending attitudes as well as time-invariant firm characteristics using firm-fixed effects. We also incorporate year and industry dummies in addition to the firm-fixed effect to control for the time-fixed effect and the industry-fixed effect (3-digit SIC). The year-fixed effect helps control for varying economic or social conditions that could influence banks’ attitudes over time. The industry-fixed effect aids in long-term control of potential changes in the industry.

Most literatures do not control both firm level fixed effect and industry fixed effect simultaneously. If a firm’s industry does not change over time, we can solely control firm-fixed effect [30], as the firm-fixed effect covers industry fixed effect. However, if the firm’s industry changes over time, we should control industry fixed effect besides the firm fixed effect. As our set of examples are large and over a long period, we need to concern about industry fixed effect. Many Chinese papers control the industry fixed effect and firm-fixed effect at the same time [31]. Therefore, we adopt a three-way fixed-effect regression model. Three-way includes firm fixed effect, industry-fixed effect and year-fixed effect.

**Fig 4:**
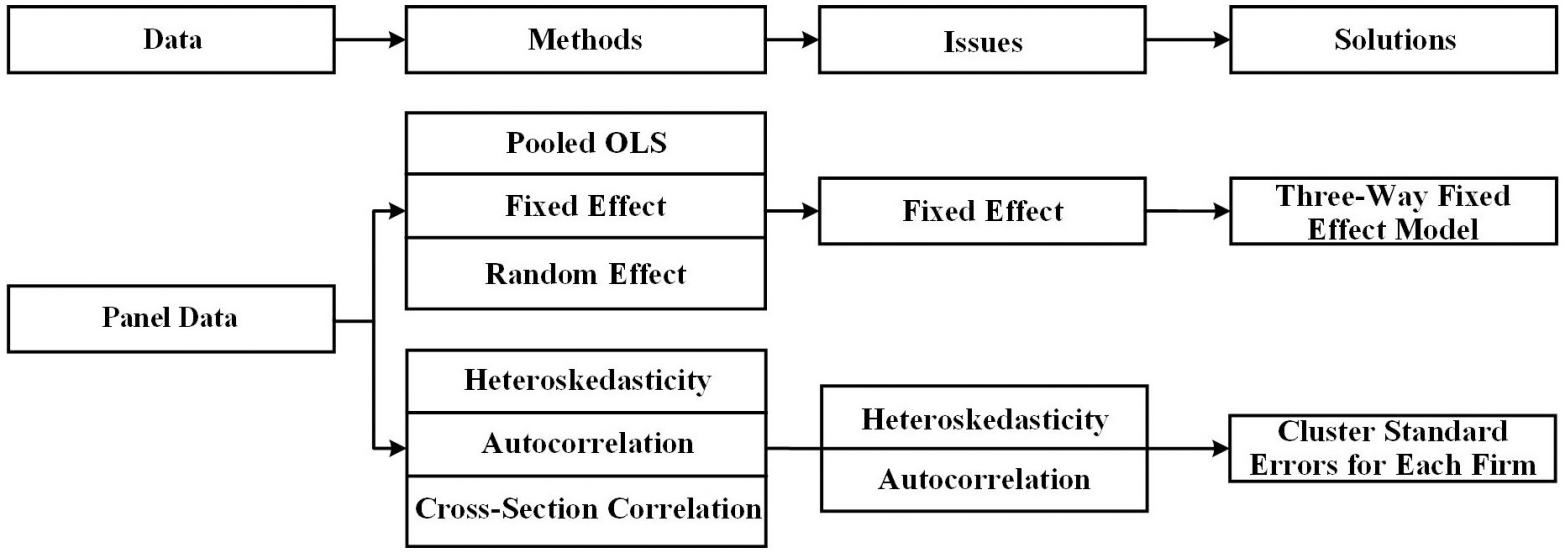
Selection of the method.

Besides the fixed effect model, we have to further test residual error heteroskedasticity, autocorrelation, and cross-sectional correlation of our panel data before running the regression. We find residual error heteroskedasticity and autocorrelation in our data but not cross-sectional correlation. In order to account for any potential autocorrelation and heteroskedasticity issues, we cluster the standard errors for each firm. The procedure for picking up those methods can be well explained in Fig 4.

### 3.4 Descriptive statistics

Table 2 presents the descriptive statistics for all variables across the sample years. Bank lending size is 20% of total assets on average. The lending cost is around 4%-6%, and the value is similar to the prior study [5]. Government grant ranges from 0 to 5%. It is 0.5% of total assets on average. Since the standard deviation of *Growth* is three times more than the mean, there are significant variations in *Growth* among the enterprises. The *ROA* has a similar condition of *Growth*.

**Table 2:** Descriptive statistics of variables.

Variables	N	Mean value	Standard deviation	25% quantile	Median	75% percentile	Minimum	Maximum value
*Loan*	15408	0.190	0.179	0.041	0.152	0.286	0	0.826
*Cost*	15408	0.060	0.112	0.000	0.042	0.068	0	0.846
*Sub*	15408	0.005	0.007	0.001	0.002	0.006	0	0.046
*PPE*	15408	0.409	0.190	0.272	0.400	0.548	0.014	0.839
*Grow*	15408	0.186	0.621	-0.030	0.080	0.229	-0.625	4.712
*Lev*	15408	0.498	0.210	0.338	0.500	0.652	0.072	1.030
*Size*	15408	22.46	1.432	21.48	22.32	23.33	19.21	26.46
*Roa*	15408	0.039	0.067	0.011	0.036	0.070	-0.245	0.235
*Own*	15408	0.607	0.488	0	1	1	0	1

Table 3 shows the results of the bivariate correlations of our study variables. The result shows that bank lending size and cost both have a positive relationship with government grants at the 10% significant level. Since the correlations among the independent variables are less than 0.4, multicollinearity should not be a concern in our study, indicating that the model can be used to do further analysis. Lind, Marchal, and Mason (2002) [41] point out that multicollinearity may exist if the correlation coefficients exceed 0.7.

**Table 3:** Correlation coefficient of the variables.

Variables	*Loan*	*Cost*	*Sub*	*PPE*	*Grow*	*Lev*	*Size*	*Roa*	*Own*
*Loan*	1	0.571***	0.013*	0.282***	0.019**	0.531***	0.247***	-0.321***	0.091***
		0.000	0.095	0.000	0.020	0.000	0.000	0.000	0.000
*Cost*	0.466***	1	0.023***	0.302***	-0.043***	0.351***	0.091***	-0.309***	0.045***
	0.000	.	0.005	0.000	0.000	0.000	0.000	0.000	0.000
*Sub*	0.001*	0.001*	1	-0.051***	-0.001	-0.149***	-0.064***	0.040***	-0.040***
	0.054	0.055	.	0.000	0.889	0.000	0.000	0.000	0.000
*PPE*	0.233***	0.301***	-0.008	1	-0.012	0.260***	0.124***	-0.181***	0.135***
	0.000	0.000	0.299	.	0.137	0.000	0.000	0.000	0.000
*Grow*	-0.001	-0.006	-0.020**	-0.023***	1	0.051***	0.113***	0.253***	-0.024***
	0.968	0.483	0.015	0.005	.	0.000	0.000	0.000	0.003
*Lev*	0.483***	0.408***	-0.064***	0.257***	0.053***	1	0.366***	-0.421***	0.172***
	0.000	0.000	0.000	0.000	0.000	.	0.000	0.000	0.000
*Size*	0.186***	0.100***	-0.115***	0.141***	0.028***	0.320***	1	0.043***	0.250***
	0.000	0.000	0.000	0.000	0.000	0.000	.	0.000	0.000
*Roa*	-0.257***	-0.276***	-0.063***	-0.137***	0.132***	-0.422***	0.103***	1	-0.150***
	0.000	0.000	0.000	0.000	0.000	0.000	0.000	.	0.000
*Own*	0.086***	0.055***	-0.021***	0.136***	-0.043***	0.164***	0.254***	-0.112***	1
	0.000	0.000	0.009	0.000	0.000	0.000	0.000	0.000	.

***, **, and * stand for correlation significant at 1%, 5%, and 10% levels.

## 4 Empirical results

### 4.1 Baseline regression results

Table 4 presents the results of the baseline regression. For all specifications, we reject the null hypothesis of the explanatory variables being jointly insignificant. *Sub* is statistically significant whether in Columns (1) and (3) without control variables or in Columns (2) and (4) with control variables. To be more specific, in Column (2), we discover that the regression coefficient of *Sub* on *Loans* is positive and statistically significant (0.505, p-value = 0.039), showing that banks would like to lend more money to the high-subsidized enterprises and supporting hypothesis H1a. The coefficient of *Sub* on *Cost* in Column (4) is positive and statistically significant (0.088, p-value = 0.007), supporting H2b’s assertion that banks charge more to high-subsidized enterprises. In conclusion, banks exhibit diverse opinions regarding the size and cost of lending.

**Table 4:** Banks’ attitudes to government grants.

	(1)	(2)	(3)	(4)	(5)	(6)
	*Loan*	*Loan*	*Loan-PSM*	*Cost*	*Cost*	*Cost-PSM*
***Sub***	0.607**	0.505**		0.120***	0.088***	
	(0.036)	(0.039)		(0.001)	(0.007)	
***Sub-Dum***			0.027*			0.002*
			(0.081)			(0.053)
*PPE*		0.014	0.048		0.024***	0.023***
		(0.441)	(0.125)		(0.000)	(0.000)
*Grow*		0.001	0.001		0.001	0.001
		(0.911)	(0.951)		(0.404)	(0.737)
*Lev*		0.344***	0.320***		0.033***	0.033***
		(0.000)	(0.000)		(0.000)	(0.000)
*Size*		0.024***	0.030***		0.001	0.001**
		(0.000)	(0.000)		(0.369)	(0.029)
*Roa*		-0.063*	-0.068		-0.015***	-0.032***
		(0.052)	(0.194)		(0.001)	(0.000)
*Own*		-0.002	-0.003		0.001	0.003***
		(0.871)	(0.879)		(0.858)	(0.003)
*Intercept*	0.190***	-0.469***	-0.675***	0.009	-0.026**	-0.022***
	(0.007)	(0.000)	(0.000)	(0.156)	(0.036)	(0.010)
*Indu FE*	Yes	Yes	Yes	Yes	Yes	Yes
*Year FE*	Yes	Yes	Yes	Yes	Yes	Yes
*Firm FE*	Yes	Yes	Yes	Yes	Yes	Yes
N	15408	15408	2104	15408	15408	2104
adj.R^2^	0.036	0.184	0.179	0.023	0.132	0.246
F	9.367	25.38	23.71	8.543	18.77	25.03

Note: Standard deviations are robust p-values adjusted for individual clustering, ***, **, and * indicate significant at 1%, 5%, and 10% levels respectively.

We further tested the impact of whether firms receive government grants on bank borrowing. As can be seen from the descriptive statistics in the previous table, the vast majority of listed companies receive government grants. If direct regression is conducted, it will lead to an imbalanced sample distribution and biased conclusions. Therefore, we use the propensity score method, using enterprise size, ownership, growth rate, profit margin, and industry to a 1:1 match sample. The regression results in Columns (3) and (6) are similar to the above, which is banks are more willing to lend money to firms that can receive government grants but charge a higher interest. This finding shows that receiving or not receiving government grants for banks’ clients could affect lending decisions.

### 4.2 Robustness tests

The above findings could be due to alternative explanations. To rule out other explanations, we run extra tests in this section. First, our results could be confounded by endogeneity issues. Endogeneity bias is fixed via instrumental variables regression. This topic will be addressed in Section 3.4.1. Second, our results result from a flawed methodology, as banks might decide not to extend any credit to certain subsidized firms, which could cause the dependent variable to have a left-tail cut. Section 3.4.2 is to demonstrate that this alternative explanation is unlikely to account for all of our findings. Third, as government grants may alter annually and cease at some point, the findings may be influenced by erroneous measurements of those grants. This alternate theory is put to the test in Section 3.4.3. Fourth, particular cities or regions may become reliant on government budgets over time. As a result, we further control the fixed provincial-year effect.

#### (1) Endogeneity and instrumental variables approach (IV-2SLS)

Although fixed effects aid in the control of missing variables, reverse causality may still affect our results. We employ an instrumental variable (IV) strategy to address this. The average government grants (Sub-Mean) of other enterprises in the same city and year, in the same city and industry, and the same city and year, respectively, are used to construct three IVs. We assume that policies enjoyed by businesses in the same industry and year, those enjoyed by businesses in the same city and the same industry, as well as the economic environment and the level of government support, will be similar. For the dependent variables to be exogenous, three instrumental factors must be connected with the independent variable. Similar to Column 1 of Table 5. Three IV coefficients in stage one are all noticeably positive. The rule-of-thumb critical value for a weak instrument test, 8.96. 70.98 in our test is above the F-statistic for testing a weak instrument, suggesting that our regression is unaffected by the weak instrument problem. The regression findings from stage two are reported in Columns (2) and (3). In line with the conclusions above, we discover that the instrumented *Sub* coefficients are positive and statistically significant.

**Table 5:** Robustness tests.

	(1)	(2)	(3)	(4)	(5)	(6)	(7)	(8)	(9)
	Stage I	Stage-II-Size	Stage-II-Cost	Tobit-Size	Tobit-Cost	Aver-Size	Aver-cost	P*Y-Size	P*Y-Cost
***Sub***		4.622***	0.409**	0.636**	0.079***	1.170**	0.197***	0.508**	0.091***
		(0.001)	(0.034)	(0.023)	(0.005)	(0.013)	(0.003)	(0.037)	(0.005)
*PPE*	0.001**	0.00300	0.023***	0.037*	0.018***	0.0310	0.022***	0.010	0.024***
	(0.060)	(0.752)	(0.000)	(0.067)	(0.000)	(0.124)	(0.000)	(0.571)	(0.000)
*Grow*	0.001***	-0.001	0.001	-0.001	-0.001	0.001	0.001	0.001	0.001
	(0.001)	(0.406)	(0.135)	(0.943)	(0.109)	(0.926)	(0.308)	(0.965)	(0.402)
*Lev*	-0.001	0.355***	0.034***	0.397***	0.020***	0.343***	0.033***	0.345***	0.033***
	(0.475)	(0.000)	(0.000)	(0.000)	(0.000)	(0.000)	(0.000)	(0.000)	(0.000)
*Size*	-0.001	0.027***	0.001***	0.034***	0.001**	0.020***	0.001**	0.024***	0.001
	(0.558)	(0.000)	(0.003)	(0.001)	(0.937)	(0.001)	(0.031)	(0.000)	(0.402)
*Roa*	0.001***	-0.039	-0.014***	-0.043	-0.023***	-0.084**	-0.014***	-0.066**	-0.015***
	(0.000)	(0.104)	(0.000)	(0.245)	(0.000)	(0.022)	(0.007)	(0.041)	(0.001)
*Own*	0.001	-0.003	0.001	-0.009	-0.001	-0.010	0.001	0.001	0.001
	0.112	(0.711)	(0.706)	(0.502)	(0.603)	(0.477)	(0.769)	(0.990)	(0.885)
***IV1***	0.005***								
	(0.001)								
***IV2***	0.005***								
	(0.000)								
***IV3***	0.007***								
	(0.000)								
*Indu FE*	Yes	Yes	Yes	Yes	Yes	Yes	Yes	Yes	Yes
*Year FE*	Yes	Yes	Yes	Yes	Yes	Yes	Yes	Yes	Yes
*Firm FE*	Yes	Yes	Yes	Yes	Yes	Yes	Yes	Yes	Yes
Intercept				-1.049***	-0.023	-0.468***	-0.045***	-0.455***	-0.025**
				(0.000)	(0.101)	(0.000)	(0.004)	(0.000)	(0.050)
N	15408	15408	15408	15408	15408	11902	11902	15408	15408
adj.	0.051	0.048	0.008	0.176	0.114	0.177	0.128	0.187	0.136
F	70.98	84.95	56.66	16.70	7.89	15.00	13.98	17.90	8.081

Note: Panel instrumental variable regressions do not report constants because individual effects have been considered, and the common constant is not meaningful.

#### (2) Truncated regression and Tobit

Variables must follow a normal distribution in order to use the OLS. However, because banks may decide not to lend money to some businesses, the dependent variables in our model might be left truncated at 0. Any regression model in the class known as Tobit models censors the observed range of the dependent variable in some way. Truncated and other non-randomly picked samples can be handled using Tobin’s method [42]. Tobin’s idea was to modify the likelihood function to reflect the unequal sampling probability for each observation depending on whether the latent dependent variable fell above or below the determined threshold. In order to build a fixed effect model, we import LSDVs (least squares dummy variables) into the Tobit model. The coefficients in the Least Square Dummy Variable model are identical to that in the fixed effect Model. As seen in Columns (4) and (5) of Table 5, the outcomes support our earlier conclusions.

#### (3) Redefine independent variable

Government budgets or policy changes could make government grants insecure, which means government grants may fluctuate broadly. This study thus substitutes the mean value of government grants over the previous three years to improve government grants’ measurement. The outcomes, displayed in Table 5’s columns (6) and (7), align with our earlier discoveries.

#### (4) Controlling for provincial-year fixed effect

Government grants, though sticky in regions, may change over the years. So, we consider the individual fixed effect of province*year instead of a single fixed effect of province and year, which could help reduce omitted variables and bias in our findings. The regression results in Columns (8) and (9) of Table 5 are consistent with our previous findings.

## 5 Mechanism exploration

The baseline regression presents a general effect of the variables, while a grouping analysis could help uncover the underneath mechanisms. Many scholars try to uncover the mechanisms by dividing the sample into different groups via corporate governance, firm size, and region. However, we counter that these kinds of grouping cannot discover the heterogeneous effects of different government grants but the effects of the same government grant under different circumstances. Additionally, much literature uses interactions rather than separate regressions to examine the impacts of heterogeneity. However, we contend that interactions are biased because they presume that all groups fit the requirements (are statistically significant), even though some groups may not (statistically insignificant).

To find heterogeneity, we, therefore, use separate regressions by groups rather than interaction. In order to analyze the underlying heterogeneous effects of our independent variables, we divide our sample into two categories of government grants rather than other unrelated factors.

Government grants are not distributed at random. They always have certain tendencies. For instance, government grants for high-tech, innovative, and high-potential firms are primarily "development supportive" subsidies, such as support for innovation. In contrast, government grants for conventional, less innovative, underperforming, and state-owned firms are primarily "helping hands" subsidies, such as funding for social responsibility and loss support. In light of this, we categorize government grants according to the relationships between their objectives and the characteristics of the firms.

### (1) High or low tech

High-tech businesses are those in emerging industries that continuously engaging in research and development. Most government grants they received were for "development supportive," such as funds for talent acquisition and research and development. Because of this, we categorize government grants based on whether the companies receiving them are high-tech or not.

As the results in Columns (1) and (2) of Table 6, Government grants (*Sub*) are significantly and positively related to bank lending (*Loan*) in high-tech firms only but not in low-tech firms. The results in Columns (1) and (2) of Table 7 show that Government grants (*Sub*) are positively related to the cost of lending (*Cost*) in low-tech firms but not in high-tech firms.

**Table 6:** Heterogenous effects of government grants on lending size.

	(1)	(2)	(3)	(4)	(5)	(6)	(7)	(8)
	Hightech	Lowtech	H-R&D	L-R&D	H-Q	L-Q	NoState-O	State-O
***Sub***	0.503*	0.470	0.563**	0.564	0.799**	0.218	0.660**	0.298
	(0.063)	(0.397)	(0.039)	(0.177)	(0.013)	(0.533)	(0.048)	(0.348)
*PPE*	0.018	-0.001	-0.001	0.049	-0.015	0.018	0.012	-0.015
	(0.468)	(0.981)	(0.968)	(0.159)	(0.506)	(0.450)	(0.643)	(0.544)
*Grow*	-0.001	0.003	0.001	-0.002	0.005*	-0.003	0.003	-0.002
	(0.697)	(0.390)	(0.823)	(0.484)	(0.081)	(0.147)	(0.217)	(0.425)
*Lev*	0.371***	0.273***	0.348***	0.374***	0.397***	0.317***	0.287***	0.425***
	(0.000)	(0.000)	(0.000)	(0.000)	(0.000)	(0.000)	(0.000)	(0.000)
*Size*	0.020***	0.039***	0.029***	-0.001	0.021***	0.028***	0.031***	0.009
	(0.001)	(0.000)	(0.000)	(0.947)	(0.001)	(0.000)	(0.000)	(0.254)
*Roa*	-0.042	-0.125**	-0.078**	-0.0730	-0.065*	-0.064	-0.043	-0.113**
	(0.260)	(0.043)	(0.030)	(0.245)	(0.081)	(0.203)	(0.338)	(0.016)
*Own*	-0.003	0.002	-0.007	0.003	-0.005	-0.015		
	(0.854)	(0.905)	(0.566)	(0.912)	(0.680)	(0.364)		
*Intercept*	-0.410***	-0.691***	-0.612***	0.054	-0.443***	-0.504***	-0.657***	-0.107
	(0.002)	(0.000)	(0.000)	(0.844)	(0.001)	(0.004)	(0.000)	(0.550)
*Indu FE*	yes	yes	yes	yes	yes	yes	yes	yes
*Year FE*	yes	yes	yes	yes	yes	yes	yes	yes
*Firm FE*	yes	yes	yes	yes	yes	yes	yes	yes
N	11427	4026	10654	4790	7704	7704	6157	9251
adj.	0.197	0.163	0.199	0.174	0.231	0.147	0.172	0.201
F	17.64	13.42	18.96	15.43	15.02	10.39	34.838	65.965
Suest	0.082*		0.094*		0.037**		0.055*	

**Table 7:** Heterogenous effects of government grants on lending size.

	(1)	(2)	(3)	(4)	(5)	(6)	(7)	(8)
	Hightech	Lowtech	H-R&D	L-R&D	H-Q	L-Q	NoState-O	State-O
***Sub***	0.041	0.252***	0.063	0.177***	0.055	0.093**	-0.021	0.140***
	(0.279)	(0.000)	(0.110)	(0.001)	(0.146)	(0.031)	(0.693)	(0.000)
*PPE*	0.028***	0.017***	0.021***	0.026***	0.010***	0.008***	0.018***	0.027***
	(0.000)	(0.000)	(0.000)	(0.000)	(0.000)	(0.007)	(0.000)	(0.000)
*Grow*	0.001	0.001	0.001	0.001	0.001	0.001	0.001	0.001
	(0.385)	(0.827)	(0.531)	(0.476)	(0.611)	(0.620)	(0.758)	(0.185)
*Lev*	0.035***	0.028***	0.034***	0.030***	0.005*	0.005*	0.032***	0.035***
	(0.000)	(0.000)	(0.000)	(0.000)	(0.077)	(0.080)	(0.000)	(0.000)
*Size*	0.001	0.001	0.001	-0.001	-0.001	-0.003***	0.001	-0.001
	(0.278)	(0.701)	(0.346)	(0.532)	(0.198)	(0.000)	(0.224)	(0.517)
*Roa*	-0.013**	-0.021**	-0.018***	-0.005	-0.024***	-0.029***	-0.022***	-0.008
	(0.014)	(0.019)	(0.001)	(0.545)	(0.000)	(0.000)	(0.001)	(0.209)
*Own*	-0.001	0.003	0.001	-0.001	-0.001	0.001		
	(0.498)	(0.511)	(0.966)	(0.840)	(0.518)	(0.895)		
*Intercept*	-0.035**	-0.013	-0.034**	0.006	0.034**	0.077***	-0.048**	-0.003
	(0.014)	(0.558)	(0.016)	(0.809)	(0.035)	(0.000)	(0.020)	(0.863)
*Indu FE*	yes	yes	yes	yes	yes	yes	yes	yes
*Year FE*	yes	yes	yes	yes	yes	yes	yes	yes
*Firm FE*	yes	yes	yes	yes	yes	yes	yes	yes
N	11427	4026	10654	4790	5992	5992	6157	9251
adj.	0.151	0.116	0.128	0.121	0.085	0.090	0.119	0.160
F	15.65	11.32	14.74	14.70	7.535	8.86	22.911	50.029
Suest	0.006***		0.029**		0.075*		0.026**	

Note: we use the *suest* to compare the coefficients across subgroups.

### (2) Innovation capability

Likewise, the government grants received in high R&D firms are mostly "development supportive" subsidies. Most literature uses R&D investment or patents to measure innovation capability, but considering the fact that some firms may not apply for patents and the presence of multiple types of patents (e.g., invention, appearance design, etc.), the number of patents may not be relevant in measuring the innovation activities of firms. Thus, we refer to R&D investment to represent innovation capability by using it to help classify government grants.

As we can see from the results in Columns (3) and (4) of Table 6, government grants (*Sub*) and bank lending size (*Loan*) are significantly and positively correlated in high R&D firms only but not in low R&D firms. The results in Columns (3) and (4) of Table 7 show that government grants (*Sub*) are positively related to the cost of lending (*Cost*) in low R&D firms only but not in high R&D firms.

### (3) High and low market value

For high-market-value firms, it is less likely that they would receive “helping hands” subsidies. Investors’ evaluation of firms can adequately measure the potential value of firms. For this reason, we adopt Tobin Q to assess the potential market value of firms and classify government grants.

From the results in Columns (5) and (6) of Table 6, government grants (*Sub*) are significantly and positively connected to bank lending size (*Loan*) in high development potential firms only, while it is insignificant in low development potential firms. In contrast, the results in Columns (5) and (6) of Table 7 show that government grants (*Sub*) are significantly and positively related to the cost of lending (*Cost*) only in low-market-value firms but not in high-market-value firms.

### (4) State-owned or nonstate-owned

According to the widely held opinion that government grants are subject to "ownership discrimination," state-owned firms are more likely to get grants due to their political ties to the government or social obligations. Chinese state-owned and state-supported firms benefit from financial assistance, regulatory privileges, and exemptions that are typically unavailable to their privately-owned rivals [43, 44]. For instance, one study [45] finds that state-owned businesses received more government grants than nonstate-owned businesses. Thus, banks may interpret the grants obtained in state-owned companies as "helping hands" and increase the interest rate on loans.

Our findings in Columns (7) and (8) of Table 6 support our hypothesis that nonstate-owned businesses receive more bank lending than state-owned businesses. Table 7’s columns (7) and (8) demonstrate that banks charge nonstate-owned businesses a lower lending cost than they do to state-owned businesses.

### (5) Discussion

We uncover evidence that banks only have a positive attitude toward businesses that are non-state-owned, high-tech, high market value and high R&D investment businesses. Government support for those businesses primarily takes the form of "development supportive", which can encourage banks. However, we do, demonstrate that government aid increases the cost of financing to low-tech, low-R&D investment, low-market value, and state-owned businesses. Government grants are primarily "helping hands" to these businesses, which could send out negative signals that prompt banks to reevaluate the possible credit risks of businesses and raise their lending risk premiums.

## 6 A further test on the mechanism

One study [46] argues that a firm’s grants could be a combination of "development supportive" grants and "helping hands" grants. We, therefore, measure two types of government grants separately in this section.

In China, a single item of "non-operating income" is used to present all government grants in the income statement. In 2017, the PRC Ministry of Finance dropped this "crude" practice by distinguishing "government grants connected to operating activities" from "government grants irrelated to operating activities" and required to report them separately (CAS16 Article 11). In other words, "government grants associated to operating activities" equivalent to "development supportive" grants, is presented in “other income”. "Government grants unrelated to operating operations", which are equivalent to "helping hand" grants, are presented in non-operating income. Separate government grants presentations could give the banks a clearer and stronger signal.

As the revision in financial presentations kicked off in 2017, we select the sample spanning from 2017 to 2019. The model and variable settings remain the same as the above. The regression results are shown in Table 8. As in Column (1), government grants are significantly and positively related to lending size (*Loan*) and negatively related to loan cost (*Cost*) in "other income" (*Sub1*). This suggests that "development supportive" grants encourage banks to lend more money and result in positive feedback. Normally, "development supportive" grants are typically accepted by banks together with operating profits. As in Column (3), "non-operating income" (*Sub2*) government grants have a significant negative correlation with lending size (*Loan*) and a significant positive correlation with cost (*Cost*), suggesting that "helping hands" grants may send the negative signals and cause banks to restrict lending and raise risk premium. The results are similar when we also consider Tobit regressions, as seen in Columns (2) and (4) of Table 8.

**Table 8:** Regression results based on presentations of government grants.

	(1)	(2)	(3)	(4)
	Size-OLS	Size-Tobit	Cost-OLS	Cost-Tobit
*Sub-income*	1.269*	1.777**	-0.640**	-0.623*
	(0.071)	(0.050)	(0.041)	(0.064)
*Sub-others*	-3.893***	-4.759***	0.744*	0.734*
	(0.000)	(0.000)	(0.058)	(0.083)
*PPE*	0.085***	0.107***	-0.004	-0.004
	(0.000)	(0.000)	(0.569)	(0.638)
*Grow*	-0.005	-0.003	0.003	0.003
	(0.280)	(0.571)	(0.277)	(0.251)
*Lev*	0.407***	0.490***	-0.008	-0.007
	(0.000)	(0.000)	(0.442)	(0.546)
*Size*	0.001	0.006	0.003***	0.003**
	(0.723)	(0.137)	(0.010)	(0.028)
*Roa*	-0.147**	-0.215**	-0.093***	-0.098***
	(0.042)	(0.032)	(0.007)	(0.005)
*Own*	-0.011*	-0.016	-0.004	-0.004
	(0.088)	(0.101)	(0.129)	(0.181)
*Intercept*	-0.025	-0.197**	0.030	0.027
	(0.708)	(0.038)	(0.200)	(0.336)
*Indu FE*	Yes	Yes	Yes	Yes
*Year FE*	Yes	Yes	Yes	Yes
*Firm FE*	Yes	Yes	Yes	Yes
N	1952	1952	1727	1727
adj.R^2^	0.318	2.303	0.036	0.016
F	40.41	42.66	3.802	5.123

In summary, the results show that banks can identify the different signals from different types of government grants and make different lending decisions.

## 7 Conclusions

Bank plays an important role in allocating financial resources to fuel economic growth. This study investigates whether banks can identify their clients’ different government grants and respond differently. In order to verify this idea, we use a unique Chinese dataset of 15408 firm-year from 2009 to 2019 and construct a three-way fixed effect model. The large sample econometric analyses highlight that different government grants would transmit different signals and result in varied bank lending attitudes. In particular, "development supportive" grants received by banks’ clients may send positive signals to banks and encourage them to lend more, whereas "helping hands" subsidies may send negative signals to banks, causing them to raise risk premiums and reduce lending. Our results are robust up well to different explanations.

Besides the contributions to the behavioural finance theory, our findings also have two implications for banks. First, rather than relying solely on financial data to make lending decisions, banks should incorporate non-financial information (e.g. Management Discussion Analysis) and indirect evidence (e.g. specific events) to help better understand clients and make sound lending decisions. Second, banks should separate clients’ different government grants instead of taking them in a general way and embracing them into lending decisions.

From a policymaker perspective, our findings suggest that the government should standardize the disclosure of government grants and fully use government grants’ signal effect on capital allocation. A better categorization, measurement, and presentation of government grants would benefit a more efficient market. The government should also utilize the signal effect of government grants to convey signals of promising industries and direct the market. In addition, the government should take into account of the unintended "spillover effects " in addition to direct "subsidy effects" when developing subsidy policies. For instance, while the "helping hand" grant can boom a firm’s short-term profit, it may also indirectly make financing for upgrading more difficult.

This study’s limitation is that we can only indirectly assess the banks’ attitudes toward government grants. This paper fails to include all other control variables in the model, despite our best efforts to tangle it up with a three-way fixed effect model. Future studies could try an experimental test to better control variables since the process of making bank lending decisions is complicated and arbitrary.

## Supporting information

S1 Appendix Definitions of variables. (DOCX)
