# Supplementary material for: The “heterogeneous” effect of government grants on bank lending
Source: PLoS One. 2023 Dec 11;18(12):e0289375. doi: 10.1371/journal.pone.0289375 (PMC10712854; doi:10.1371/journal.pone.0289375)
Supplement: S1 Appendix — (DOCX) [file pone.0289375.s001.docx]

# Appendix A

Definitions of Variables

| Variables | Names | Definitions |
| --- | --- | --- |
| *Loan* | Lending size | Cash received from borrowings/total ending assets |
| *Cost* | Lending costs | Interest expense/total ending interest-bearing liabilities |
| *Sub* | Government grant | Government grant/total ending assets |
| *PPE* | Tangible assets percentage | (Ending fixed assets + Ending inventories)/Total ending assets |
| *Grow* | Sales growth | (Current sales - prior sales)/prior sales |
| *Lev* | Financial leverage | Total liabilities/total assets |
| *Size* | Firm size | Natural logarithm of total ending assets |
| *Roa* | Return on assets | Net profit/total ending assets |
| *Own* | Dummy variable | 1 for state-owned enterprises, 0 for others |
